# Next-Generation of Allergen-Specific Immunotherapies: Molecular Approaches

**DOI:** 10.1007/s11882-018-0790-x

**Published:** 2018-06-09

**Authors:** Mirela Curin, Musa Khaitov, Alexander Karaulov, Leyla Namazova-Baranova, Raffaela Campana, Victoria Garib, Rudolf Valenta

**Affiliations:** 1 Division of Immunopathology, Department of Pathophysiology and Allergy Research, Center for Pathophysiology, Infectiology and Immunology, Medical University of Vienna, Waehringer Guertel 18-20, 1090 Vienna, Austria; 2 NRC Institute of Immunology FMBA of Russia, Moscow, Russia; 3 Laboratory of Immunopathology, Department of Clinical Immunology and Allergy, Sechenov First Moscow State Medical University, Moscow, Russia; 4 Scientific Centre of Children’s Health, Moscow, Russia; 5 International Network of Universities for Molecular Allergololgy and Immunology, Vienna, Austria

**Keywords:** Allergy, Allergen, Molecular allergology, Allergen-specific immunotherapy (AIT), Recombinant allergen, Hypoallergens, Peptides, Allergen-specific prevention of allergy, Peptide-carrier vaccine

## Abstract

**Purpose of Review:**

The aim of this article is to discuss how allergen-specific immunotherapy (AIT) can be improved through molecular approaches. We provide a summary of next-generation molecular AIT approaches and of their clinical evaluation. Furthermore, we discuss the potential of next generation molecular AIT forms for the treatment of severe manifestations of allergy and mention possible future molecular strategies for the secondary and primary prevention of allergy.

**Recent Findings:**

AIT has important advantages over symptomatic forms of allergy treatment but its further development is limited by the quality of the therapeutic antigen preparations which are derived from natural allergen sources. The field of allergy diagnosis is currently undergoing a dramatic improvement through the use of molecular testing with defined, mainly recombinant allergens which allows high-resolution diagnosis. Several studies demonstrate that molecular testing in early childhood can predict the development of symptomatic allergy later on in life.

**Summary:**

Clinical studies indicate that molecular AIT approaches have the potential to improve therapy of allergic diseases and may be used as allergen-specific forms of secondary and eventually primary prevention for allergy.

## Introduction

Immunoglobulin E (IgE)-associated allergy is the most common immunologically mediated hypersensitivity disease [1, 2]. It affects more than 30% of the population world-wide and represents a heavy burden for the patients and health care systems [3]. Allergic patients suffer from a variety of symptoms such as rhinitis, conjunctivitis, skin inflammation, gastrointestinal symptoms, asthma, and life-threatening systemic anaphylactic reactions. Allergic symptoms are typically triggered by the recognition of environmental antigens (i.e., allergens) by IgE antibodies and the subsequent activation of inflammatory cell responses by allergen-IgE immune complexes [4••]. Using serum samples collected from children after birth, during childhood, and up to adolescence in birth cohorts, it has become possible to analyze the evolution of allergen-specific IgE responses and to establish associations between IgE sensitization profiles and the subsequent development of allergic symptoms [5–10, 11•, 12••]. Certain forms of allergy such as food allergy and atopic dermatitis develop very early in childhood, whereas respiratory forms of allergy such as rhinitis and conjunctivitis start often with a silent IgE sensitization (i.e., production of allergen-specific IgE antibodies without symptoms) and then progress from mild to severe manifestations of disease. Available data from birth cohorts strongly suggest that molecular allergy diagnosis is not only useful for the diagnosis of allergy but allows to predict the development of allergic diseases based on the measurement of IgE sensitization profiles early in life [7••, 12••]. It has been shown that AIT performed in childhood can prevent the progression of mild forms of allergy to severe forms of allergy and thus may be useful as a kind of secondary prevention of allergy [13, 14]. Accordingly, it has been suggested to investigate if AIT can be used in secondary and primary allergen-specific preventive approaches [15, 16]. At present, AIT is mainly used for the treatment of allergic rhinoconjunctivitis, venom allergy, and for certain forms of food allergy mainly because of limitations through the poor and varying quality of therapeutic allergen preparations from natural allergen sources [17]. However, it seems that this problem can be overcome by the use of defined molecular vaccines. Here, we argue that only next-generation molecular allergy vaccines will make it possible to substantially improve AIT in its current indications, to expand the use of AIT to severe forms of allergy (e.g., asthma) and for the secondary and eventually primary prevention of allergy [15, 18•, 19, 20].

## The Development of AIT Toward Molecular Vaccines

AIT is the administration of vaccines comprising the disease-causing allergens to allergic patients with the goal to induce an immune response which protects the patient against allergic inflammation upon allergen contact. The induction of allergen-specific IgG antibodies which interfere with the interaction between specific IgE antibodies and allergens is a major mechanism underlying AIT besides effects on a variety of immune cells (e.g., T cells, APCs, mast cells, eosinophils) [21, 22]. Three major mechanisms mediated by allergen-specific IgG antibodies have been demonstrated for AIT. First, AIT-induced allergen-specific IgG antibodies bind allergen and prevent it from binding and cross-linking of mast cell and basophil-bound IgE and subsequent immediate allergic inflammation [21]. Co-cross-linking of IgE and IgG receptors on mast cells with subsequent silencing of mast cell activation has been considered as additional mechanism but support for the latter comes mainly from experimental animal studies, whereas it is not sure if this mechanism is important for humans [23, 24]. Second, AIT-induced allergen-specific blocking IgG antibodies interfere with IgE-facilitated allergen-presentation to T cells and thus reduce allergen-specific T cell activation and inflammatory cytokine production [25, 26••]. Thus, allergen-specific IgG reduces T cell-mediated allergic inflammation and most probably also eosinophil activation. Third, it was found that boosts of allergen-specific IgE production upon allergen encounter is reduced in patients who had received AIT and produced allergen-specific IgG, and it has been suggested that AIT-induced allergen-specific IgG can prevent the activation of allergen-specific IgE production [26••, 27–29, 30••]. The latter mechanism may play an important role for the long-term effects of AIT several years after discontinuation but also sustained allergen-specific IgG4 levels may be important in this context [31, 32].

**Fig. 1:**
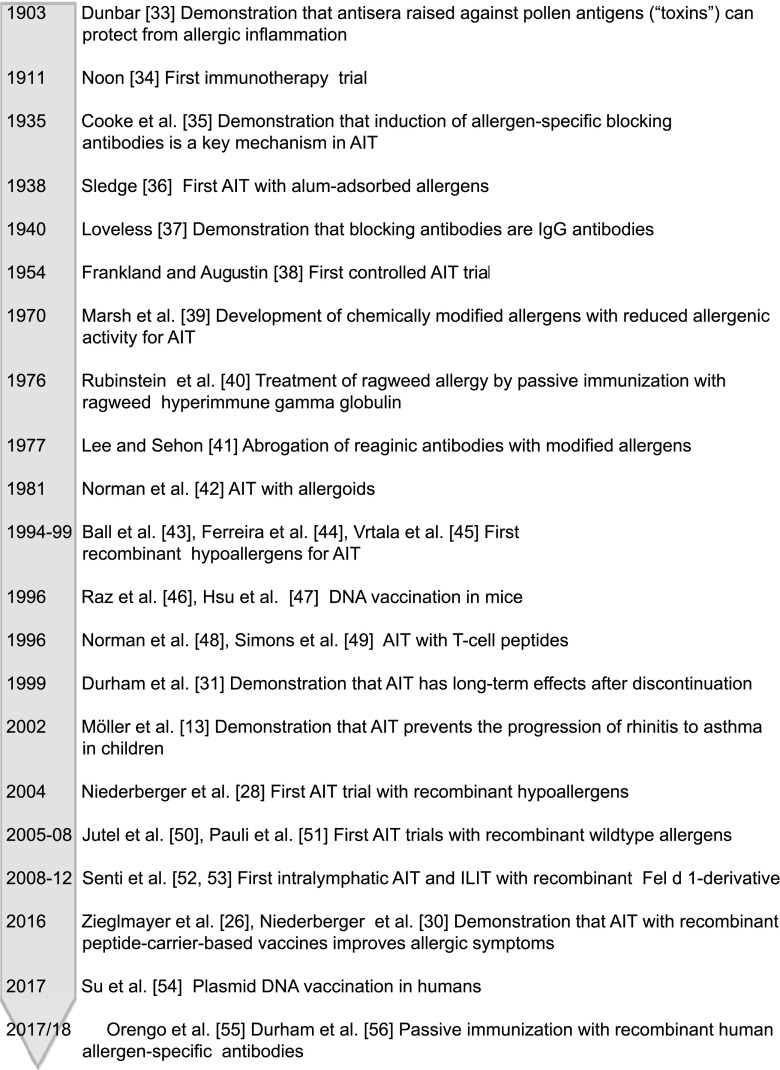
Time line showing some highlights of AIT toward molecular AIT

The time line in Fig. 1 summarizes some advances in the course of AIT leading to molecular forms of AIT and corresponding references [13, 26, 28, 31, 33–53, 54•, 55••, 56]. The importance of allergen-specific IgG antibodies for the success of AIT has already been demonstrated very early. In fact, Dunbar’s publication in the year 1903 showing that antisera raised against pollen antigens prevented allergen-induced inflammation in allergic patients has been quoted by Leonard Noon in his classic first paper describing AIT for the first time [33, 34]. Dunbar has thus provided evidence that passive immunization with allergen-specific IgG can prevent allergen-induced allergic inflammation [33]. This finding was confirmed in an elegant study by Cooke who demonstrated that AIT induces allergen-specific IgG and that this allergen-specific IgG could be transferred with serum from AIT-treated patients and blocked allergen-specific skin inflammation [35]. Further work by Mary Loveless revealed that the blocking antibodies induced by AIT are IgG antibodies [37]. Three further studies from the USA and Europe showed that a reduction of allergic symptoms was achieved in patients given hyper immunoglobulin preparations which contained allergen-specific IgG antibodies [40, 57, 58]. Similar findings were also made by investigators from Stavropol in Russia who showed that treatment with immunoglobulin improved symptoms of ragweed allergy and in other studies [59, 60–62]. These early findings stimulated the idea that one can treat allergy by passive immunization with allergen-specific human IgG antibodies [63, 64], and this concept was realized in a recent clinical study which demonstrated that cat allergic patients can be successfully treated with two recombinant human IgG4 antibodies specific for the major cat allergen Fel d 1 [55••, 56].

The first AIT study by Leonard Noon was conducted with aqueous grass pollen extracts and patients experienced considerable side effects [34]. Subsequent attempts to increase the immunogenicity by the use of adjuvants led to AIT with allergen extracts which had been adsorbed to Aluminum hydroxide which currently is the most frequently used adjuvant in AIT [36]. Adsorption of allergen extracts to Aluminum hydroxide not only increased the immunogenicity of AIT and the levels of AIT-induced IgG antibodies but also contributed to improved safety. In fact, allergens which are adsorbed to Alum remain at the injection side and thus cause much less systemic side effects as compared to aqueous allergen extracts which rapidly distribute in the body and hence frequently induce severe systemic side effects [65, 66]. Another attempt to reduce allergenic side effects during AIT was the modification of allergen extracts by chemical denaturation or by conjugation with polyethylenglycol (PEG) as described by David Marsh and Alec Sehon, respectively [39, 41]. Chemically denatured allergen extracts with reduced allergenic activity, termed allergoids, were shown to be clinically effective in first published immunotherapy studies such as the one reported by Norman et al. 1981 [42]. A milestone for AIT was the development of well-controlled study protocols. In fact the first controlled AIT trial was published by Frankland and Augustin in 1954 [38]. Two important AIT trials should be mentioned which highlighted important advantages of AIT over symptomatic pharmacotherapy. The AIT study published by Stephen Durham and colleagues in 1999 showed that beneficial clinical effects are maintained even after discontinuation of treatment indicating long-term clinical effects of treatment [31]. Another study conducted by Möller and colleagues demonstrated that AIT but not anti-inflammatory pharmacotherapy can prevent the progression of rhinitis to asthma in allergic children providing evidence for a secondary preventive effect of AIT [13].

The development of molecular forms of AIT started soon after the first allergen-encoding DNA sequences were obtained by molecular cloning and the first recombinant allergen molecules became available [1]. One approach for molecular treatment was based on synthetic allergen-derived peptides targeting the receptors of allergen-specific T cells with the goal to induce T cell tolerance. The administration of synthetic T cell epitope-containing peptides of the major cat allergen Fel d 1 was shown to induce tolerance in a murine model [67]. First clinical studies with synthetic T cell epitope-containing peptides were published in the mid-1990s [48, 49]. Another approach was based on the generation of recombinant hypoallergenic allergen-derivatives which exhibited reduced IgE reactivity, preserved allergen-specific T cell epitopes, and upon immunization induced allergen-specific blocking IgG antibodies [45, 68, 69]. The first immunotherapy trial with recombinant hypoallergens in allergic patients was published by Niederberger and colleagues in 2004 [28]. One year later, the first study reporting AIT with recombinant grass pollen allergens was reported [50] and a study demonstrating that AIT performed with recombinant Bet v 1 allergen for a period of 2 years was as effective as birch pollen allergen extract or natural Bet v 1 in reducing symptoms of birch pollen allergy was published in 2008 [51].

In 2008, the first study was published which reported intralymphatic administration as a possible new route for AIT [52] and in 2012, intralymphatic administration of recombinant major cat dander allergen Fel d 1 fused to a translocation sequence (TAT) and to part of the human invariant chain, generating a modular antigen transporter (MAT) vaccine (MAT-Fel d 1) for targeting the MHC II pathway was reported [53]. In 2016, the first clinical study showing safety, effects, and immunological mechanisms of a recombinant B cell epitope-based grass pollen allergy was published [26••]. Hypoallergenic recombinant B cell epitope-based vaccines are peptide-carrier-based fusion proteins consisting of non-allergenic allergen peptides derived from the IgE binding sites of allergens and a non-allergenic carrier protein providing T cell help for the production of IgG antibodies targeting the IgE epitopes of the allergen [70•]. In 2017, the first data showing that passive immunization with two recombinant human monoclonal IgG4 specific for the major cat allergen, Fel d 1, reduces allergic symptoms of cat allergy have been reported [55••, 56]. These results are important for at least two reasons: First of all, they provide important insight into the mechanisms of AIT by demonstrating that allergen-specific blocking IgG antibodies alone are sufficient for the treatment of allergy, a finding which is also supported by the results obtained with active B cell epitope-based allergy vaccines that mainly induces allergen-specific blocking IgG [26••, 30••]. Second, the successful treatment with allergen-specific IgG is notable in light of the early demonstrations that allergen-specific antisera [33] and allergen-specific hyper-immunoglobulin preparations protected against allergic symptoms [40, 57, 58]. In 2017, another interesting study was published which reported safety and effects of vaccination with allergen-encoding plasmid DNA [54•]. In fact, immunization with allergen-encoding plasmid DNA for treatment of allergy in murine models has been reported first in 1996 [46, 47] but safety concerns arose when it was found that this treatment causes a wide distribution of allergen-encoding transcripts in the body of treated mice [71]. For the latter reason, vaccination with allergen-encoding plasmid DNA and RNA has not reached clinical application for a long time and instead oligonucleotides were used as adjuvants in molecular allergy vaccines [29].

## Advantages of AIT and Limitations of Allergen Extract-Based AIT

As outlined in Table 1, AIT has important advantages over all existing and currently emerging forms of allergy treatment. Only AIT modifies the course of the disease and appears to prevent the progression toward severe disease manifestations. Only AIT has long-term effects even after discontinuation. Importantly, AIT is a very cost-effective treatment and functions like a therapeutic vaccine by utilizing the body’s own immune system. However, all current forms of allergen-extract-based AIT have severe limitations which are due to the poor quality of the therapeutic antigens obtained from natural allergen sources. Natural allergen extracts strongly vary in their composition, are undefined regarding most of their ingredients, and can induce allergic reactions upon administration. For the latter reason, it is very unlikely that a profound improvement of AIT and the production of innovative AIT vaccines fulfilling the regulatory requirements of modern vaccines and drugs can be achieved with allergen extract-based approaches. Another potential disadvantage of AIT is that its accurate prescription requires a profound medical education in allergology and the ability to identify the disease-causing allergens by appropriate diagnostic methods. Thus AIT does not fit the desire of the big pharmaceutical industry for a “one fits it all” form of treatment.

**Table 1:** Advantages of AIT and limitations of current allergen extract-based forms of AIT

Advantages of AIT	
• The only disease-modifying allergy treatment in contrary to anti-inflammatory medications that only temporarily reduces symptoms	
• Decreases the risk of progression of the disease to more severe forms (e.g., from rhinitis to asthma)	
• Long-term effect since the beneficial outcome may last for years after treatment is completed	
• More cost effective than medication treatment	
Limitations of current allergen extract-based forms of AIT	
• Undefined mixture of allergenic and non-allergenic components	
• Natural allergen sources which are used for extract production may be limiting and allergen contents cannot be influenced	
• Important allergens are sometimes absent from the extract	
• Variable content of allergens depending on the manufacturer and even batch to batch variations from same manufacturer	
• May contain contaminations from other sources (e.g., house dust mites in animal dander extracts or endotoxins)	
• Numerous injections over long time period what causes poor compliance of patients	
• Immediate and late side effects due to fully IgE reactive natural allergens	

## Molecular Forms of AIT

**Fig. 2:**
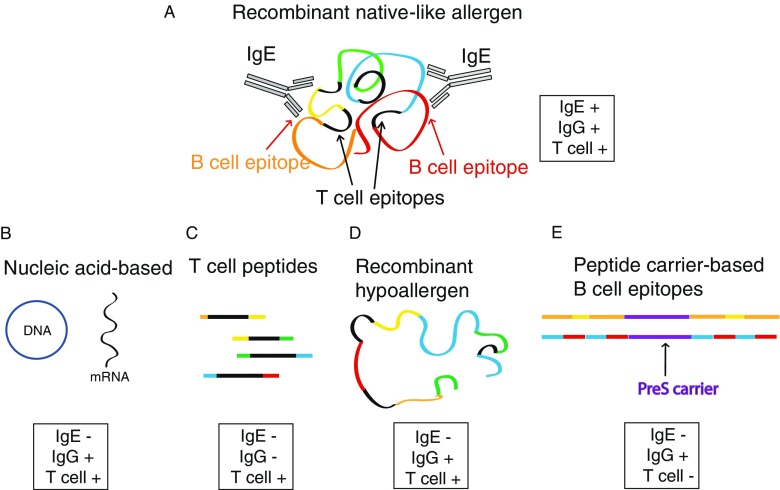
Molecular strategies for AIT

Figure 2 provides an overview of molecular forms of AIT which have entered clinical evaluation [1, 72]. The identification of the DNA and amino acid sequences of important allergens by cDNA cloning approaches beginning from 1988 has paved the road toward several strategies of AIT. These strategies include the use of recombinant native-like allergens, approaches based on genetic vaccination with allergen-encoding DNA or RNA, the use of synthetic allergen-derived peptides containing T cell epitopes, the generation of recombinant hypoallergenic allergen molecules and recently recombinant peptide carrier-based vaccines which fuse non-allergenic B cell epitope-containing peptides of allergens with a non-allergenic carrier protein providing T cell help.

### T Cell Epitope-Containing Peptides

The first molecular approach for AIT which reached clinical application was the use of synthetic allergen peptides containing T cell epitopes. This approach was initiated by ImmuLogic Pharmaceutical Corp. (Waltham, Mass., USA) [73] soon after the DNA sequence of the major cat allergen, Fel d 1, was revealed [74]. In the first clinical studies two synthetic, 27 amino acid long T cell peptides derived from cat allergen Fel d 1 were administered [48, 49]. The rationale of this approach was to use short peptides which are not IgE-reactive but can induce T cell tolerance in allergic patients. The T cell epitope-containing peptides lacked IgE reactivity but were too short to induce allergen-specific IgG antibodies upon administration. Treatment with four injections showed only modest effect [48] in reducing lung and nasal symptoms when applied in the highest dose and no improvement was reported in another study [49]. Moreover, treated patients often experienced adverse side effects in both studies. In another attempt performed by the company Circassia, a different set of Fel d 1-peptides was used by applying eight subcutaneous high-dose injections. Although treated patients had some beneficial effects regarding allergic symptoms, the treatment was often accompanied by side effects like late asthmatic reactions [75, 76]. Based on this experience, further attempts to improve the T cell peptide approach were undertaken and seven synthetic T cell epitope containing peptides with length of 13–17 amino acids were applied in cat allergic patients [77]. While there were some beneficial effects for treated patients vs placebo observed in phase II [78], a large phase III field study did not achieve clinical end points since active groups did not differ from placebo (http://www.circassia.com/media/press-releases/circassia-announces-top-line-results-from-cat-allergy-phase-iii-study/). Clinical studies based on the same principle with synthetic peptide immuno-regulatory epitopes (SPIRE) were performed in addition to cat for house dust mite, ragweed, and grass pollen allergy and the results of phase II show good safety and some beneficial effects for the patients. However, after the disappointing results of the phase III cat study, the T cell peptide approach was not further pursued.

### Recombinant Hypoallergens

The second molecular approach which reached clinical evaluation and the first which was based on recombinant molecules was performed with recombinant hypoallergenic derivatives of the major birch pollen allergen, Bet v 1 [45, 68]. Recombinant hypoallergenic allergen derivatives can be produced by recombinant expression using different technologies for reducing IgE-reactivity and allergenic activity of the allergen while at the same time T cell epitopes and immunogenicity (i.e., ability to induce allergen-specific IgG responses) are maintained [69, 79]. The technologies include fragmentation [45], oligomerization [68], mutation of residues [80, 81], creation of mosaic molecules [82], allergen hybrids [83, 84], and by displaying allergens on the virus-like particles [85]. In 2000, the first AIT trial with recombinant allergen derivatives was conducted and in this trial, hypoallergenic recombinant fragments of rBet v 1 and rBet v 1 trimer were tested in 124 birch pollen allergic patients in a double-blind, placebo-controlled study [28]. This treatment was shown to have clinical effects, to induce allergen-specific blocking IgG antibodies, reduced seasonal boosts of IgE production, and eliminated IgE-mediated immediate adverse effects. However, because most Bet v 1 T cell epitopes were maintained in the vaccine, systemic late-phase adverse effects were observed despite eliminated IgE reactivity in the immunogens [28].

An approach which is based on long T cell epitope-containing peptides of Bet v 1 (contiguous overlapping peptides: COPs) and thus was similar to the recombinant Bet v 1 fragments [28, 86] was tested in clinical studies up to phase IIb. These studies reported that birch allergic patients treated with “AllerT” vaccines over a period of 3 years experienced improvement in allergic symptoms. However, again late-phase reactions were observed [87] similarly like in the past with rBet v 1 fragments which were only slightly longer than COPs [88]. These studies indicated that the presence of allergen-specific T cell epitopes in a vaccine can give rise to T cell-mediated, IgE-independent late-phase side effects. Recently, a first vaccine for fish allergy based on subcutaneous injections of an aluminum-hydroxid-adsorbed hypoallergenic Cyp c 1 mutant was tested in humans in a phase 1/2a, in a double-blind, placebo-controlled clinical study [81]. Results showed low level of adverse effects and induction of allergen-specific IgG antibodies (clinicaltrials.gov Identifier: NCT02017626). In addition to recombinant production of hypoallergenic allergen derivatives, it was also tried to produce a recombinant hypoallergenic version of the major birch pollen allergen Bet v 1 by chemical modification of the recombinant wild-type allergen [89]. Treatment with this folding variant of Bet v 1 showed a reduction of clinical symptoms and the induction of allergen-specific IgG responses in clinical trials in patients [90] but this approach was not further pursued due to difficulties to establish a robust good manufacturing practice (GMP)-conform production process for the folding variant.

### Nucleic Acid-Based Vaccines

DNA- and mRNA-based allergy vaccines represent another molecular approach for AIT. In experimental animal models, these vaccines were shown to induce an immunological bias that prevents Th2 sensitization and drives immune response toward Th1 via induction of IFN-γ, IgG2a, and suppression of IgG1, IgE, and allergic lung inflammation [91]. It was hoped that nucleic acid-based vaccines have a reduced risk of inducing severe side effects such as anaphylaxis. mRNA-based vaccines were even considered more safe than DNA vaccines since only the genetic information of the allergen itself has to be used, whereas DNA vaccines introduce foreign sequences and might integrate into patient’s genome and propagate [92]. In one of the first studies, Roy et al. were able to demonstrate that administration of DNA nanoparticles composed of plasmid DNA that coded for peanut allergens Ara h 2 and of polysaccharide chitosan resulted in transduced gene expression in the intestinal epithelium of mice. Mice immunized with nanoparticles showed a reduction in allergen-induced anaphylaxis associated with reduced levels of IgE, plasma histamine, and vascular leakage when compared with non-immunized mice or mice treated with naked DNA [93]. These results indicated prophylactic utility of DNA vaccination for treating food allergy. Although promising, most of the findings with nucleic acid-based vaccines were derived only from animal models. Only recently, a phase I study was performed in humans, which had studied safety and long-term immunological effects of CryJ2-LAMP plasmid vaccine in Japanese red cedar atopic subjects. Published data suggested that the CryJ2-LAMP DNA vaccine is safe and may be immunologically effective in treating JRC-induced allergy [54•].

### CpG-Conjugated Allergens

Conjugating immunostimulatory sequences of DNA (e.g., CpG motifs from microbes) to allergens offers another approach utilizing the effects of DNA for AIT. The immunostimulatory sequences bind to toll-like receptor 9 (TLR9), which is expressed within endosomes of human plasmacytoid dendritic cells (pDC) and B cells and thus should promote Th1 responses. Furthermore, coupling of these sequences to allergens seemed to reduce the allergenic activity of allergens [94]. Amb a 1, the major ragweed-pollen allergen, was conjugated to a CpG immunostimulatory sequence and tested in a phase II trial and it was demonstrated that a once-weekly, six injections regiment induced clinical benefit in rhinitis patients lasting through two ragweed seasons without severe adverse events [29]. However, the limitation of this study was the small number of patients enrolled. A study with a larger number of patients (TOLAMBA study) followed but was terminated early since interim data showed that subjects exhibited no meaningful allergic disease during the first ragweed season, making it impossible to measure treatment effect (https://www.clinicaltrials.gov/ct2/show/NCT00387738). A single center, open-label phase I/IIa clinical study tested the safety and tolerability of virus-like particles (VLP) that contained packaged CpG together with a house-dust mite (HDM) allergen extract and the clinical outcome was positive when six subcutaneous injections at intervals of 1–2 weeks were applied [95].

### Recombinant Wild-Type Allergens

Recombinant allergens can be produced in high purity (GMP conditions), large quantity (milligram amounts), and consistent quality in bacteria, yeast, insect cells, and mammalian cells [96–98]. Since recombinant allergens mimic biochemically and immunologically natural allergens, they could replace allergen extracts in AIT. The first clinical trial with recombinant wild-type allergens in AIT, namely the double-blind, placebo-controlled study by Jutel et al., investigated the utility of a mixture of the wild-type form of Aluminum hydroxide-adsorbed five recombinant timothy grass allergens (Phl p 1, 2, 5a, 5b, 6) when injected subcutaneously in allergic patients. The outcomes indicated that active group of patients developed allergen-specific IgG1 and IgG4 antibody responses and an approximately 35% reduction in symptoms and medication usage was observed [50]. Another clinical study in birch pollen allergic patients compared the safety and efficacy of recombinant Bet v 1, purified natural Bet v 1, and birch pollen extract with placebo over a period of 2 years. The results showed approximately 50% reduction of rhinoconjunctivitis symptoms in the three active groups compared to placebo [51]. Interestingly, rBet v 1 was equally effective as birch pollen extract for subcutaneous immunotherapy. Nevertheless, rBet v 1 is a fully IgE-reactive protein, and therefore patients had to follow the same inconvenient up-dosing schedule and monthly maintenance injections as is used for allergen extracts to avoid adverse effects. rBet v 1 was also tested in clinical trials by applying via sublingual route in the tablet form (clinicaltrials.gov Identifier: NCT00396149). The mean adjusted symptom scores were significantly decreased relative to placebo in patients receiving once-daily rBet v 1 tablets for 5 months [99]. However, more than 70% of the patients from the actively treated group experienced local reactions in the mouth and throat due to the application of IgE-reactive Bet v 1 allergen.

### Recombinant Peptide Carrier-Based AIT

The latest generation of allergy vaccines was developed to improve the technology of recombinant hypoallergens [100, 101]. It has been demonstrated that recombinant hypoallergens containing allergen-specific T cell epitopes can elicit late phase, IgE-independent, T cell-mediated side effects [102]. Accordingly, the presence of allergen-specific T cell epitopes is reduced in the B cell epitope-containing vaccines which are based on the peptide carrier concept. T cell help to induce IgG antibodies against allergen peptides derived from the IgE binding sites of the allergens is obtained by a non-allergenic carrier protein [70•]. The feasibility of the technology was demonstrated initially with allergen peptides which were coupled chemically to keyhole limpet hemocyanin (KLH) [103–106]. In order to produce vaccines acceptable for human use which can be produced in a highly reproducible manner under good manufacturing practice conditions (GMP), carrier proteins derived from viruses were used which have already been used for vaccinations of humans (e.g., the hepatitis surface antigen, PreS) and instead of chemical coupling, recombinant fusion proteins consisting of the carrier protein and non-allergenic peptides are produced by recombinant expression [18•, 107–109, 110•]. The recombinant B cell epitope-based vaccines have several advantages. First, they lack IgE reactivity and allergenic activity since ~ 30 amino acid long peptides without fold are used as building blocks and accordingly do not induce IgE-mediated immediate adverse events [18•]. Importantly, the vaccines do not boost allergen-specific IgE responses and hence seem to be useful also for prophylactic administration because their sensitization potential seems to be low [26••]. The second advantage of these vaccines is the strong reduction of allergen-specific T cell responses since T cell help comes from allergen-independent carrier (hepatitis B virus Pre S protein) [110•, 111]. Thus not only immediate but also late phase side effects are reduced. Third, the use of a carrier protein renders the vaccines highly immunogenic so that IgG responses can be induced even against per se weakly immunogenic allergens [112•] and targets specifically the IgE binding sites on the allergens [108]. As a result, recombinant B cell vaccines can be administered in few high-dose injections without inducing relevant side effects which should achieve higher convenience and compliance of the patients to treatment. The grass pollen vaccine BM32 developed in Vienna [110•] was the first vaccine that was tested in a series of clinical trials (clinicaltrials.gov Identifiers: NCT01350635, NCT01538979, NCT01445002, and NCT02643641). The vaccine contains non-IgE-reactive peptides which are derived from the IgE-binding sites of the four most important allergens of grass pollen (Phl p 1, Phl p 2, Phl p 5, and Phl p 6) and were covalently linked to a viral protein carrier (Pre S from hepatitis B virus) that provides carrier-specific T cell help [18•, 110•]. Skin study of BM32 reported that vaccine is safe since no allergenic activity was observed when applied by skin prick- and atopy patch testing to grass pollen allergic patients [111]. Results of the double-blind, placebo-controlled phase 2b multicenter field study reported good tolerability, no severe adverse effects, and beneficial effect in the form of relief of allergy symptoms [26••, 30••]. As a carrier protein in BM32 vaccine, hepatitis B-derived PreS protein was used, and immunotherapy with BM32 also induced antibody responses that protected against hepatitis B infection in vitro what also indicates that BM32 could be useful for therapeutic vaccination of patients infected with hepatitis B virus [113••, 114]. Based on peptide carrier principle, vaccines for house dust mite, cat, and ragweed have been and are currently being developed. Furthermore, the B cell epitope-based vaccines are considered for the use as prophylactic vaccines to achieve secondary and primary allergen-specific prevention by a vaccination approach [15, 70•, 115].

## New Indications for AIT Which May Become Possible with Next-Generation Molecular Allergy Vaccines

The detailed analysis of the evolution of allergen-specific IgE sensitization with micro-arrayed allergen molecules in birth cohorts indicates that allergic sensitization develops early in life and thus defines windows of opportunity for secondary and primary preventive allergen-specific interventions in childhood [10]. Accordingly, several molecular approaches for allergen-specific prophylaxis can be considered [15]. Primary preventive interventions include the induction of immunological tolerance by administration of tolerogenic peptides or allergen derivatives before allergic sensitization has taken place [116•], early vaccination to start allergen-specific IgG responses which may prevent allergic sensitization [115], and the induction of tolerance by cell therapy early in infancy [117, 118•, 119]. Secondary prevention may be considered at two levels: First, one may consider early AIT to prevent the progression of mild forms of disease such as rhinitis toward severe forms such as asthma as has been shown in AIT studies already [13, 14]. Second, one may consider to perform AIT in children with yet silent IgE sensitizations to prevent the development of allergic disease later in life. In fact, several studies indicate that serological diagnostic tests based on micro-arrayed allergen molecules can be used to predict the development of allergic disease by early blood testing and thus to identify children who will benefit from preventive AIT [7••, 8, 9, 11•, 12••]. Table 2 summarizes some of the exciting possibilities for using AIT in the future for difficult forms of disease and prevention. However, it must be clear that defined new-generation molecular AIT approaches which can selectively target certain immunological mechanisms, which are safe and do not induce IgE sensitizations, are needed to achieve these goals (Table 2).

**Table 2:** New indications for AIT and corresponding requirements for new-generation vaccines

New indications	
• Treatment of severe forms of allergy (e.g., asthma, atopic dermatitis, food allergy)	
• Secondary prevention in childhood: Prevention of progression from mild to severe forms (e.g., rhinitis-asthma)	
• Secondary prevention in childhood: Prevention of progression from clinically silent IgE sensitization to symptomatic allergy	
• Primary prevention: Prevention of IgE sensitization in early childhood by early vaccination of not yet sensitized children or mothers to transfer protective antibodies to children	
Requirements for new-generation vaccines to comply with new indications	
• Comprise all clinically relevant allergens	
• High safety: Lack of IgE- and T cell-mediated side effects	
• Non-sensitizing, lack of allergenicity	
• Induction of high levels of allergen-specific protective IgG responses	
• Convenient application schedules to improve the compliance of patients	

## Conclusion

The elucidation of the primary sequences of the most relevant allergen molecules that began in the late 1980s has formed the basis for next-generation forms of allergen-specific immunotherapy (AIT) based on molecular approaches (Fig. 1). These new approaches may overcome the limitations of current forms of AIT because they are not any more dependent on the unpredictable composition and poor quality of natural allergen extracts. However, molecular approaches must comprise the clinically important allergen molecules of the respective allergen sources. Three major molecular AIT approaches have been evaluated (Fig. 2). The use of non-allergenic peptides containing T cell epitopes for inducing T cell tolerance was so far not effective for the treatment of established allergy most likely because this approach does not induce protective allergen-specific IgG antibodies which block IgE binding to the allergen and/or because established allergy cannot be any more controlled by interventions at the T cell level. However, it is quite possible that T cell epitope-based approaches are effective for preventing allergic sensitization in a prophylactic approach. Vaccination with allergen-encoding nucleic acids has developed only slowly because of concerns that they may induce uncontrolled synthesis of allergens in the body which may cause allergic reactions. AIT with pure recombinant allergens mimicking the natural wild-type allergens and with recombinant hypoallergenic allergen derivatives has been shown to induce allergen-specific blocking IgG antibodies and clinical effects but at higher doses may induce side effects and hence will require thoughtful up-dosing and maintenance schedules for treatment. The last generation of hypoallergenic carrier-bound B cell epitope-containing vaccines seems to allow overcoming the problem of side effects; it allows the administration injections of high doses, induces robust allergen-specific IgG responses, does not induce allergic sensitization, and hence holds great promise for revolutionizing AIT and may be even used for prophylactic vaccination against allergy.
